# Antiretroviral therapy initiation and retention among clients who received peer-delivered linkage case management and standard linkage services, Eswatini, 2016–2020: retrospective comparative cohort study

**DOI:** 10.1186/s13690-022-00810-9

**Published:** 2022-03-09

**Authors:** Duncan MacKellar, Thabo Hlophe, Dawud Ujamaa, Sherri Pals, Makhosazana Dlamini, Lenhle Dube, Chutima Suraratdecha, Daniel Williams, Johnita Byrd, James Tobias, Phumzile Mndzebele, Stephanie Behel, Ishani Pathmanathan, Sikhathele Mazibuko, Endale Tilahun, Caroline Ryan

**Affiliations:** 1 Division of Global HIV and TB, Center for Global Health, U.S. Centers for Disease Control and Prevention, Atlanta, GA USA; 2 Eswatini Ministry of Health, Mbabane, Eswatini; 3 ICF International, Atlanta, GA USA; 4 Population Services International, Mbabane, Eswatini; 5 U.S. Centers for Disease Control and Prevention, Pretoria, South Africa; 6 U.S. Centers for Disease Control and Prevention, Mbabane, Eswatini

**Keywords:** Cohort studies, Linkage to care, Retention, Africa, HIV care continuum, Treatment

## Abstract

**Background:**

Persons living with HIV infection (PLHIV) who are diagnosed in community settings in sub-Saharan Africa are particularly vulnerable to barriers to care that prevent or delay many from obtaining antiretroviral therapy (ART).

**Methods:**

We conducted a retrospective cohort study to assess if a package of peer-delivered linkage case management and treatment navigation services (CommLink) was more effective than peer-delivered counseling, referral, and telephone follow-up (standard linkage services, SLS) in initiating and retaining PLHIV on ART after diagnosis in community settings in Eswatini. HIV-test records of 773 CommLink and 769 SLS clients aged ≥ 15 years diagnosed between March 2016 and March 2018, matched by urban and rural settings of diagnosis, were selected for the study. CommLink counselors recorded resolved and unresolved barriers to care (e.g., perceived wellbeing, fear of partner response, stigmatization) during a median of 52 days (interquartile range: 35—69) of case management.

**Results:**

Twice as many CommLink than SLS clients initiated ART by 90 days of diagnosis overall (88.4% vs. 37.9%, adjusted relative risk (aRR): 2.33, 95% confidence interval (CI): 1.97, 2.77) and during test and treat when all PLHIV were eligible for ART (96.2% vs. 37.1%, aRR: 2.59, 95% CI: 2.20, 3.04). By 18 months of diagnosis, 54% more CommLink than SLS clients were initiated and retained on ART (76.3% vs. 49.5%, aRR: 1.54, 95% CI: 1.33, 1.79). Peer counselors helped resolve 896 (65%) of 1372 identified barriers of CommLink clients. Compared with clients with ≥ 3 unresolved barriers to care, 42% (aRR: 1.42, 95% CI: 1.19, 1.68) more clients with 1–2 unresolved barriers, 44% (aRR: 1.44, 95% CI: 1.25, 1.66) more clients with all barriers resolved, and 54% (aRR: 1.54, 95% CI: 1.30, 1.81) more clients who had no identified barriers were initiated and retained on ART by 18 months of diagnosis.

**Conclusions:**

To improve early ART initiation and retention among PLHIV diagnosed in community settings, HIV prevention programs should consider providing a package of peer-delivered linkage case management and treatment navigation services. Clients with multiple unresolved barriers to care measured as part of that package should be triaged for differentiated linkage and retention services.

**Supplementary Information:**

The online version contains supplementary material available at 10.1186/s13690-022-00810-9.

## Background

To achieve HIV epidemic control by 2030, countries throughout the world are striving to diagnose 95% of persons living with HIV (PLHIV), initiate and retain on antiretroviral therapy (ART) 95% of diagnosed PLHIV, and virally suppress 95% of PLHIV retained on ART (95–95–95) [1]. In sub-Saharan Africa, the region with the greatest burden of HIV with an estimated 67% of 37.7 million PLHIV worldwide, achieving and sustaining 95–95–95 is expected to help reduce by 78% the annual number of new HIV infections, from an estimated 861,000 in 2020 to 193,000 in 2030 [1, 2].

To help initiate and retain on ART ≥ 90% of all PLHIV as part of the 95–95–95 initiative, many sub-Saharan African countries have implemented a mix of community-based HIV testing strategies to diagnose those who otherwise might not test in clinical settings [1, 3]. PLHIV diagnosed in community settings in sub-Saharan Africa who usually are not seeking healthcare when tested, however, are particularly vulnerable to many real and perceived barriers to HIV care (e.g., perceived wellbeing, fear of partner response, stigmatization) [4–11]. When provided referral as the only linkage service, few (< 50%) enroll in care within 90 days of diagnosis, and exceptionally few initiate ART within 7 days of diagnosis as recommended by the World Health Organization (WHO) [12–21]. Two studies in Eswatini (formerly Swaziland), a country in sub-Saharan Africa with an estimated HIV prevalence of 27.3% among adults aged 15–49 years, suggest that one-third or fewer community-diagnosed PLHIV enroll in HIV care within 6 months of diagnosis in the absence of linkage services [14, 15, 22].

In 2014 and 2016, peer-delivered linkage and retention services were recommended by the U.S. Centers for Disease Control and Prevention (CDC) and WHO, respectively, to help clients avoid or resolve barriers to early ART initiation and retention [23, 24]. Few studies, however, have evaluated the efficacy of peer-delivered services to resolve barriers and improve early ART initiation and retention, and findings from these studies have been mixed [25]. To improve early ART initiation among community-diagnosed PLHIV in Eswatini, we implemented a peer-delivered linkage case management program (CommLink), based in part on early outcomes of a similar program in Tanzania [26, 27]. CommLink HIV-positive peer counselors provided a comprehensive package of CDC and WHO recommended linkage services during an average two-month case management period [3, 23, 24, 27].

A program evaluation of CommLink found that nearly all clients received recommended services, counselors helped resolve a majority of identified barriers, and during test and treat when all patients were eligible for ART, ≥ 90% of clients across demographic groups initiated ART [27]. The evaluation, however, did not include a control group and did not assess ART retention after the first antiretroviral refill. Notably, at the end of case management, 36% of CommLink clients had ≥ 1 unresolved barriers to care and may have been at high risk for discontinuing ART [27]. Although the program evaluation suggested that CommLink was highly effective in linking clients to care, the efficacy of CommLink to initiate *and* retain clients on ART compared with contemporary peer-delivered standard linkage services in Eswatini remained unknown.

To address this question, we report findings from a retrospective study on ART initiation and retention comparing clients diagnosed in community settings in Eswatini who received CommLink services with those who received peer-delivered standard linkage services (SLS). Among CommLink clients, we also report the association between unresolved barriers to care and losses to the ART initiation and retention (HIV-care) cascade. Our findings may be important for programs and countries considering the scale up of CDC and WHO recommended peer-delivered services and how these services might be improved to help countries achieve 95–95–95.

## Methods

### Time period and location

**Fig. 1:**
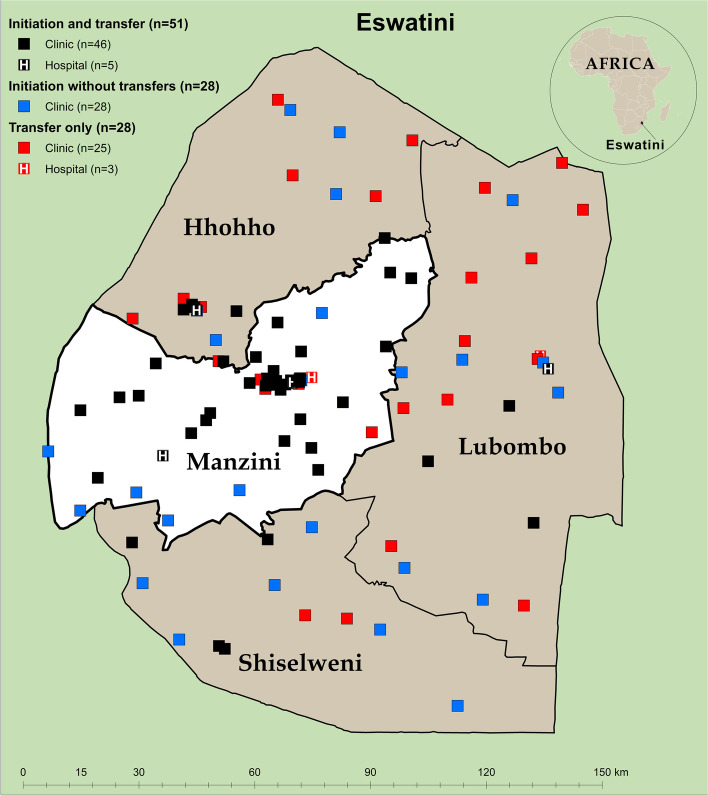
Healthcare facilities (*n*  =  107) where clients who received CommLink and peer-delivered standard linkage services ever received antiretroviral therapy after HIV diagnosis in community settings in Manzini Region, Eswatini, March 2016 – April 2020. * ART* antiretroviral therapy, *Initiation facility* healthcare facility where clients received ART after HIV diagnosis, *Transfer facility* healthcare facility where clients transferred ART care after ART initiation, *Initiation without transfers*, healthcare facilities where clients initiated ART, but did not transfer ART care, *Transfer only* healthcare facilities where clients only received ART after initiation at a different facility, *Clinic* includes health centers and public health units

Between 1 March 2016 and 31 March 2018, Population Services International (PSI) CommLink and SLS teams conducted rapid HIV testing in accordance with national guidelines at homesteads, worksites, bars, and other community locations in Manzini region, Eswatini (Fig. 1) [28]. In two urban Tinkhundla (similar with districts) of Manzini, CommLink teams did outreach testing from 1 March 2016 to 30 September 2016 when patients with a CD4 count ≤ 500/μL were eligible for ART. SLS teams did outreach testing in these urban Tinkhundla 1 October 2016 to 31 March 2018 during test and treat, when all PLHIV were eligible for ART. In the 13 rural Tinkhundla of Manzini, both CommLink and SLS teams did outreach testing during test and treat, 1 October 2016 to 31 March 2018 (see Supplementary Table 1, Additional file 1).

### CommLink and SLS services

Depending on the outreach team, clients who tested HIV positive and consented for follow-up services met with HIV-positive peer counselors who provided either linkage case management (CommLink), or standard counseling, referral, and telephone follow-up in accordance national guidelines (SLS) (Table 1) [27–29]. CommLink services, provided for up to 90 days for most clients, included point-of-diagnosis HIV medical assessment and peer-delivered counseling, at least two follow-up face-to-face HIV counseling and psychosocial-support sessions focused on identifying and resolving barriers to care, escort and treatment navigation services during at least the first healthcare visit, weekly telephone support and appointment reminder calls, and HIV testing of partners and family members (Table 1). CommLink counselors used a standard form to record barriers to care identified during client-centered counseling, and those considered resolved and unresolved by the end of case management (see Supplementary Table 2, Additional file 1) [27].

**Table 1:** Comparison of peer-delivered linkage case management (CommLink) and peer-delivered standard linkage services (SLS)

Services	CommLink^a^	SLS^b^
Point-of-diagnosis peer-delivered HIV psychosocial support, counseling on the importance of early enrollment in HIV care and ART initiation, and referral to a healthcare facility appropriate to meet client needs. HIV-positive peer counselors of both programs received the same Ministry of Health training on HIV/AIDS, and providing psychosocial support and ART-adherence counseling	Routine	Routine
Point-of-diagnosis HIV medical services including CD4 testing, WHO staging, and provision of 7-day supply of cotrimoxazole. Community-based ART initiation was not approved by the Eswatini Ministry of Health and was never provided by CommLink nurses	Routine	Never
Follow-up telephone calls to assess well-being and coping, answer questions about HIV/AIDS, encourage and support enrollment and retention in care, remind clients of upcoming appointments, coordinate treatment navigation (CommLink only), and schedule and coordinate testing of sexual partners and family members (CommLink only)	Weekly through end of case management	At least once 3–5 days after HIV diagnosis
One-time free transportation services to referral healthcare facility	Upon request	Upon request
Personal escort and peer-delivered treatment-navigation services at facilities where clients enrolled in HIV care. CommLink peer counselors stayed with their client for the duration of at least their first healthcare visit, and most met their clients at a second or third visit to confirm antiretroviral refills. As part of treatment navigation, they introduced clients to facility-based ART adherence counselors and healthcare staff, and ensured they understood the stations and sequence of care and when and how best to access care at specific facilities. Peer counselors also provided adherence counseling for cotrimoxazole and ART and helped affect transfers to other facilities when needed	Routine	Upon request
At least two follow-up peer-delivered face-to-face sessions to provide psychosocial support and informational, motivational, and ART-adherence counseling; assess and resolve real and perceived barriers to care; and support disclosure to and testing of partners and family members when safe and appropriate	Routine	Never
Peer-delivered assessment and resolution of real and perceived barriers to enrollment and retention in care. Peer counselors used a standard form to record up to 13 different barriers to enrollment or retention in HIV care (CommLink only). Identification and resolution of real and perceived barriers was conducted as part of client-centered counseling	All sessions and during telephone follow-up	First session and telephone follow-up
Index-client testing services to support disclosure and facilitate HIV testing of partners and family members when safe and appropriate	Peer- and HTS-counselor supported	HTS-counselor supported

*SLS* peer-delivered standard linkage services, *HTS* HIV testing and counseling

^a^Provided a comprehensive package of peer-delivered CDC and WHO recommended linkage services during an average two-month case management period [3, 23, 24, 27]

^b^Provided peer-delivered counseling, referral, and telephone follow-up services in accordance national guidelines [28]

### Study eligibility and selection of HIV-test records

**Fig. 2:**
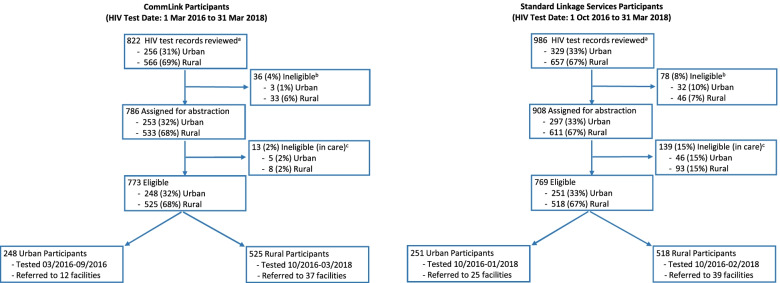
Selection of archived HIV test records of clients who received CommLink and peer-delivered standard linkage services**.** *Tinkhundla* geopolitical regional subdivisions of Eswatini**.** ^a^Population Services International (PSI) clients who tested HIV-positive during community-outreach events conducted in two urban and 13 rural Tinkhundla of Manzini region; all archived records of PSI clients who tested HIV-positive in Manzini region 1 March 2016 to 31 March 2018 were accessed and reviewed for eligibility**.** ^b^Did not meet any of the following conditions: aged ≥ 15 years, had not received HIV care in the prior 90 days, consented to follow-up linkage services, and referred for HIV care in any healthcare facility in Manzini region or in regional border zones**.** ^c^Medical record indicating client had received HIV care in the 90 days before their PSI test date

CommLink and SLS clients aged ≥ 15 years who tested HIV-positive and had not received HIV care in the prior 90 days, consented for follow-up services, and were referred to healthcare facilities in Manzini or in regional border zones were eligible for the study. All archived HIV-test records of CommLink and SLS clients who were diagnosed in Manzini region between 1 March 2016 and 31 March 2018 were accessed and reviewed for eligibility. All eligible CommLink and an approximate matching number of eligible SLS HIV-test records were selected for the study, by urban or rural location of diagnosis (Fig. 2). For urban Tinkhundla, all SLS eligible records were selected in chronological order of test date beginning with the oldest month and year when SLS teams replaced CommLink (October 2016), and ending when a matching number of eligible SLS records were identified to replace those of clients found to be ineligible at abstraction (January 2018). In rural Tinkhundla, all eligible SLS records were selected to replace ineligible records to achieve an approximate matching sample. Based on findings of a prior retrospective study in Eswatini, we anticipated that > 7% of SLS clients with an eligible archived record would be ineligible at abstraction because they did not report at diagnosis that they had received HIV care within the past 90 days [15]. Few CommLink clients were expected to be ineligible because case management and treatment navigation helped identify clients who were reluctant to disclose receiving HIV care at the time of their test.

### Matching and abstraction of medical records

**Fig. 3:**
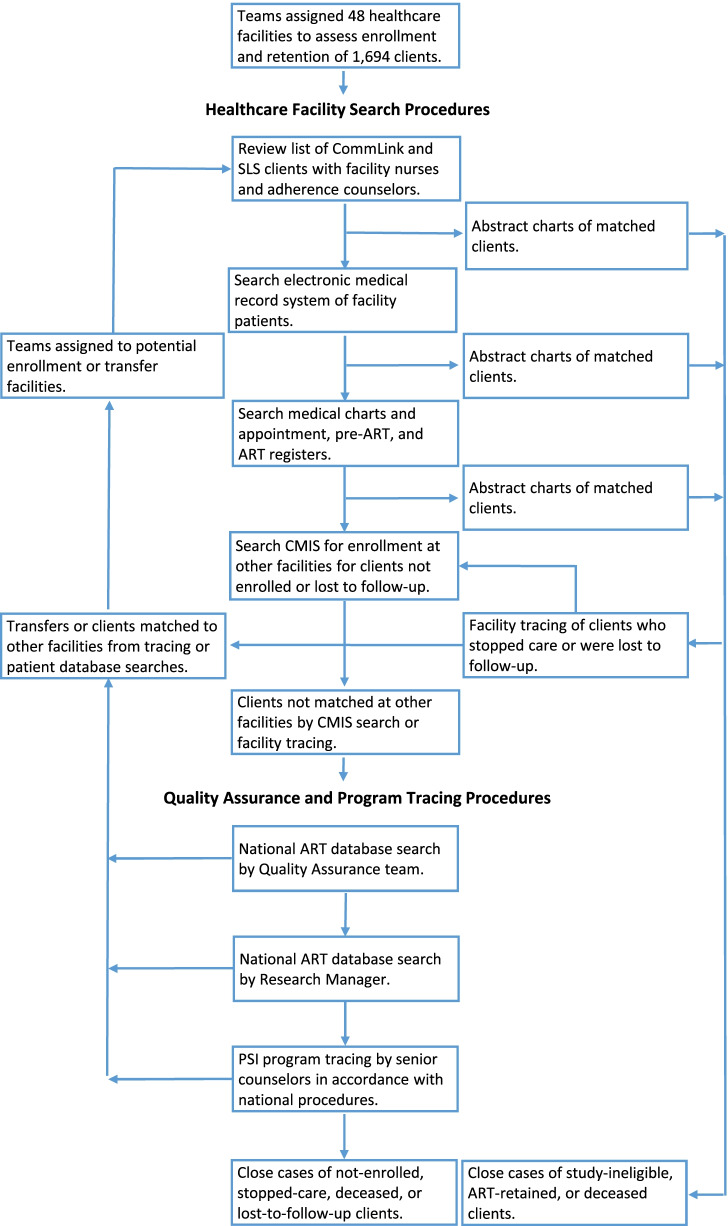
Study search, data-abstraction, and case-closure algorithm. *SLS* peer-delivered standard linkage services, *ART* antiretroviral therapy, *CMIS* Client Management Information System (national patient medical record database)

Beginning 9 July 2019, abstraction teams visited referral facilities recorded on selected HIV-test records and used all available electronic and paper-based medical records or registers, as needed, to locate, match, and abstract clinical data of study clients (Fig. 3). Nearly all facilities visited had an electronic medical record system, usually the national client management information system (CMIS). At least a three-variable match was required on sex, first or last name, and at least one other personal identifying information (e.g., date of birth, residential address, telephone number). A standard form was used to abstract clinical outcomes from matching medical records (see Supplementary Table 3, Additional file 1). If available, the patient’s HIV medical chart was the data source for abstraction; otherwise, electronic medical records or registers were used.

### Tracing clients lost to follow-up

Facility staff were informed of all enrolled clients who were not retained on ART so that tracing could be conducted in accordance with national procedures [28]. For clients not initiated or retained on ART, teams routinely searched CMIS for other facilities where they might have enrolled or transferred care. Quality-assurance staff and the study manager, separately, also searched the national ART database for all clients who were not initiated or retained on ART. Searches were conducted manually and with a computer-based probabilistic search algorithm (see Supplementary Table 4, Additional file 1). Finally, using client information on HIV-test records, PSI program staff attempted to contact all remaining clients who were lost to follow-up to assess where they might have received HIV care. Contacted clients who reported not receiving HIV care were referred for care and offered transportation and treatment-navigation services. If CMIS or national database searches, or tracing by healthcare or PSI-program staff suggested clients either enrolled in or transferred care to a different facility in Eswatini, data abstraction teams would visit those facilities to search for, match, and abstract data in accordance with the above methods (Fig. 3). All study tracing, data abstraction, and quality assurance activities were completed 30 April 2020.

### Outcome definitions

ART initiation was defined as receiving ART at least once on or after the PSI test (HIV diagnosis) date. Retained on ART was defined as not being more than 90 days late for the last antiretroviral refill appointment on the date of abstraction [28]. Lost to follow-up was defined as not retained on ART. In accordance with WHO and Eswatini HIV care and treatment guidelines, viral load suppression was defined as having < 1,000 HIV RNA copies/mL [24, 28].

### Statistical analysis

Demographic characteristics of CommLink and SLS clients were compared using Rao-Scott Chi-square tests, adjusting for within-Tinkhundla clustering. ART initiated, and combined initiated and retained on ART, were evaluated at defined time points after HIV diagnosis for all clients up to a maximum of 18 months for the combined outcome. We used generalized estimating equations (GEE) models (SAS 9.4) with a log link to estimate outcomes, relative risks, and 95% confidence intervals for ART initiated, combined initiated and retained on ART, viral load suppression, transfer of ART care, death, and lost to follow-up. Separate GEE models estimating ART initiated within 90 days of diagnosis, and initiated and retained on ART by 18 months of diagnosis, were also fit for clients by sex, age, and geographic subgroups, and for Commlink clients by barriers-to-care subgroups (no barriers identified, all barriers resolved, and 1–2 and ≥ 3 remaining unresolved barriers). Time to ART initiation and time retained on ART were estimated with Kaplan–Meier methods using SAS, and an accelerated failure-time parametric survival model using STATA (retention only). We estimated ART retention probabilities at different timepoints and 95% confidence intervals using STATA STREG postestimation commands, censoring lost-to-follow-up clients on their last antiretroviral appointment date. GEE and parametric survival models adjusted for age, sex, and geographic area, and within-Tinkhundla clustering.

### Ethical considerations

The study was approved by the Eswatini Health Research Review Board, the PSI Research Ethics Board, and the CDC Institutional Review Board. In accordance with U.S. 45CFR 46.116 (d), a waiver of informed consent for the abstraction of clinical data was approved because the evaluation was retrospective, involved no more than minimal risk, and would not adversely affect the rights and welfare of clients.

## Results

Of 1,808 archived records reviewed of clients who tested HIV-positive, 1694 (94%) were eligible and assigned for data abstraction. During July 2019 – April 2020, abstraction teams visited 129 healthcare facilities, and at 112 facilities matched 1,423 clients with 1,885 HIV medical records (median matching variables 5, interquartile range (IQR) 4—6). Based on medical-record abstractions, 13 (2%) CommLink and 139 (15%) SLS clients received HIV care in the 90 days before their PSI test date (median 27, IQR 7—51) and were re-classified as ineligible (Fig. 2).

Of 1,542 eligible clients, proportionally more CommLink than SLS clients were men, aged ≥ 35 years, married or co-habitating, and referred to clinics rather than hospitals (Table 2). Of 1,263 clients who enrolled in care, 1,208 (96%) had a CD4 cell count or WHO stage recorded at their pre-ART enrollment or ART-initiation visit (baseline). Baseline CD4 counts were similar between CommLink (median 393/µL, IQR 232—617) and SLS (median 380/µL, IQR 234—577) clients, and similar low proportions of both groups had a baseline CD4 count < 200/µL or WHO Stage III or IV disease (Table 2).

**Table 2:** Characteristics of clients who received CommLink and peer-delivered standard linkage services, by urban and rural area of HIV diagnosis, Manzini region, Eswatini, March 2016 – April 2020

	**Urban**		**Rural**		**Combined**		
**Characteristics**	**CommLink** **n (%)**	**SLS** **n (%)**	**CommLink** **n (%)**	**SLS** **n (%)**	**CommLink** **n (%)**	**SLS** **n (%)**	***p*-value**^**a**^
Total	248 (100)	251 (100)	525 (100)	518 (100)	773 (100)	769 (100)	
Sex							< 0.0001
Men	154 (62.1)	107 (42.6)	285 (54.3)	212 (40.9)	439 (56.8)	319 (41.5)	
Women	94 (37.9)	144 (57.4)	240 (45.7)	306 (59.1)	334 (43.2)	450 (58.5)	
Age group (years)							< 0.0001
15–24	34 (13.7)	62 (24.7)	80 (15.2)	121 (23.4)	114 (14.7)	183 (23.8)	
25–34	104 (41.9)	118 (47.0)	233 (44.4)	245 (47.3)	337 (43.6)	363 (47.2)	
≥ 35	110 (44.4)	71 (28.3)	212 (40.4)	152 (29.3)	322 (41.7)	223 (29.0)	
Marital status^b^							0.0048
Single	130 (60.2)	193 (77.2)	318 (65.2)	375 (72.4)	448 (63.6)	568 (74.0)	
Married/cohabitating	86 (39.8)	57 (22.8)	170 (34.8)	143 (27.6)	256 (36.4)	200 (26.0)	
Referral facility^c^							0.0406
Hospital	15 (6.0)	38 (15.1)	35 (6.7)	36 (6.9)	50 (6.5)	74 (9.6)	
Clinic, HC, or PHU	233 (94.0)	213 (84.9)	490 (93.3)	482 (93.1)	723 (93.5)	695 (90.4)	
Advanced HIV disease^d^							0.6569
No	175 (72.9)	123 (75.0)	400 (78.7)	234 (79.1)	575 (76.9)	357 (77.6)	
Yes	65 (27.1)	41 (25.0)	108 (21.3)	62 (20.9)	173 (23.1)	103 (22.4)	
ART-eligibility period^e^							–
CD4 ≤ 500/µL	241 (97.2)	0	2 (0.4)	0	243 (31.4)	0	
Test and treat	7 (2.8)	251 (100)	523 (99.6)	518 (100)	530 (68.6)	769 (100)	

*SLS* peer-delivered standard linkage services, *HC* health center, *PHU* public health unit, *ART* antiretroviral therapy, *Tinkhundla* geopolitical regional subdivisions of Eswatini

^a^Rao-Scott Chi-square, adjusting for clustering within urban and rural Tinkhundla

^b^32 urban and 37 rural CommLink clients, and 1 SLS urban client, had missing information on marital status

^c^57 facilities including 2 hospitals, 50 clinics, 4 health centers, and 1 public health unit

^d^CD4 count < 200/μL or diagnosed with WHO Stage III or IV disease at enrollment in HIV care; of 1263 clients enrolled in care, 1208 (96%) had either a CD4 count or WHO stage recorded at their enrollment or ART-initiation visit

^e^National guidelines recommending ART based on CD4 count were expanded during CommLink, resulting in the following two ART-eligibility periods: 1 March 2016 to 30 September 2016 (patients with CD4 count ≤ 500/μL) and 1 October 2016 to 31 March 2018 (all patients with any CD4 count, test and treat)

**Fig. 4:**
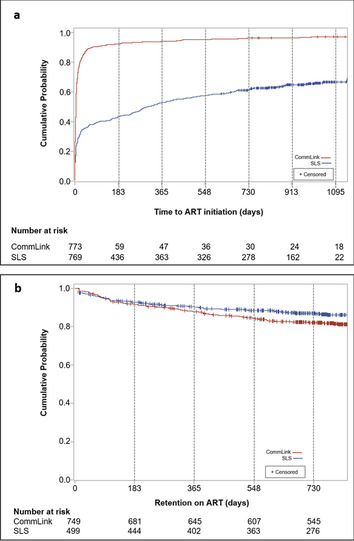
Kaplan–Meier estimates of time from HIV diagnosis in community settings in Manzini region to facility-based antiretroviral therapy initiation (**a**), and time retained on antiretroviral therapy after initiation (**b**), by CommLink and peer-delivered standard linkage service cohorts, Eswatini, March 2016 – April 2020. *SLS* peer-delivered standard linkage services, *ART* antiretroviral therapy

CommLink and SLS clients initiated ART a median of 5 (IQR 1—15) and 306 (IQR 8—unknown) days after diagnosis, respectively (Fig. 4a). By 90 days of diagnosis, over twice as many CommLink than SLS clients initiated ART overall, and among all sex and age groups (Table 3, Table 4). In rural areas during test and treat, 96.2% of CommLink and 37.1% of SLS clients initiated ART within 90 days of diagnosis. In urban areas during the CD4 ≤ 500 ART-eligibility period for CommLink and test and treat for SLS, 78.6% of CommLink and 43.9% of SLS clients initiated ART within 90 days of diagnosis. Overall, 96.6% of CommLink and 64.3% of SLS clients ever initiated ART at 79 facilities during a median follow-up period of 961 (IQR 827—1093) days (Table 3, Fig. 1, Fig. 4a).

**Table 3:** Study outcomes of clients who received CommLink and peer-delivered standard linkage services, Manzini region, Eswatini, March 2016 – April 2020

	**Unadjusted**		**Adjusted**^**a**^			
**Outcomes**	**CommLink** **n (%)**	**SLS** **n (%)**	**CommLink** **% (95% CI)**	**SLS** **% (95% CI)**	**RR (95% CI**)	***p*-value**
ART initiation^b^	*n* = *773*	*n* = *769*				
7 days	486 (62.9)	192 (25.0)	61.9 (56.6, 67.8)	23.9 (18.8, 30.5)	2.59 (1.98, 3.38)	< 0.0001
30 days	644 (83.3)	264 (34.3)	80.1 (76.7, 83.5)	32.7 (27.0, 39.5)	2.45 (1.99, 3.01)	< 0.0001
90 days	699 (90.4)	302 (39.3)	88.4 (86.6, 90.1)	37.9 (32.1, 44.7)	2.33 (1.97, 2.77)	< 0.0001
Ever^c^	749 (96.9)	499 (64.9)	96.6 (95.6, 97.7)	64.3 (57.2, 72.4)	1.50 (1.33, 1.70)	< 0.0001
Transfer ART care^d^	*n* = *749*	*n* = *499*				
Ever^c^	123 (16.4)	75 (15.0)	17.3 (15.6, 19.3)	14.6 (13.1, 16.3)	1.19 (1.02, 1.38)	0.0280
ART retention^a,e^	*n* = *749*	*n* = *499*				
6 months	(93.2)	(94.5)	93.4 (92.2, 94.6)	95.0 (93.5, 96.4)	0.98 (0.97, 0.999)	0.0328
12 months	(89.7)	(91.7)	89.9 (88.3, 91.5)	92.3 (90.3, 94.3)	0.97 (0.95, 0.998)	0.0338
18 months	(86.9)	(89.4)	87.1 (85.1, 89.1)	90.2 (87.7, 92.6)	0.97 (0.93, 0.998)	0.0343
24 months	(84.5)	(87.5)	84.8 (82.5, 87.0)	88.3 (85.6, 91.1)	0.96 (0.92, 0.997)	0.0346
Viral suppression^f^	*n* = *566*	*n* = *359*				
Ever	546 (96.5)	347 (96.7)	95.2 (94.7, 95.7)	96.7 (95.9, 97.6)	0.98 (0.98, 0.99)	0.0004
Last test	536 (94.7)	343 (95.5)	93.7 (92.2, 95.2)	95.4 (94.0, 96.9)	0.98 (0.97, 0.99)	0.0019
ART initiated and retained^b^	*n* = *773*	*n* = *769*				
6 months	650 (84.1)	308 (40.1)	80.2 (77.0, 83.6)	38.4 (33.4, 44.1)	2.09 (1.78, 2.45)	< 0.0001
12 months	630 (81.5)	362 (47.1)	79.0 (76.4, 81.8)	46.0 (40.4, 52.4)	1.72 (1.49, 1.98)	< 0.0001
18 months	612 (79.2)	388 (50.5)	76.3 (73.0, 79.7)	49.5 (44.0, 55.7)	1.54 (1.33, 1.79)	< 0.0001
Other	*n* = *773*	*n* = *769*				
Deceased^g^	22 (2.8)	14 (1.8)	2.4 (1.6, 3.8)	1.7 (0.8, 3.5)	1.44 (0.92, 2.27)	0.1111
Lost to follow-up	173 (22.4)	331 (43.0)	22.8 (18.8, 27.7)	42.5 (38.8, 46.5)	0.54 (0.42, 0.69)	< 0.0001

*SLS* peer-delivered standard linkage services, *ART* antiretroviral therapy, *RR* relative risk, *CI* confidence interval, *Tinkhundla* regional geopolitical subdivisions of Eswatini

^a^Estimated using generalized estimating equations (GEE) models with a log link (SAS 9.4), excluding ART retention; ART retention and relative risks were estimated with accelerated failure-time parametric survival models using STATA STREG postestimation commands, censoring lost-to-follow-up clients on their last antiretroviral appointment date; GEE and parametric survival models adjusted for age group, sex, urban or rural area of HIV diagnosis, and within-Tinkhundla clustering

^b^After date of HIV diagnosis among all CommLink and SLS clients

^c^Median follow-up period 961 days (interquartile range 827—1093)

^d^Receipt of ART at ≥ 1 different healthcare facilities in Eswatini after ART initiation

^e^Among clients initiated on ART, retained is defined as not being more than 90 days late for the last antiretroviral refill appointment

^f^Among clients initiated on ART who had at least one documented viral load test: 566 (75.6%) CommLink and 359 (71.9%) SLS ART-initiated clients had at least one documented viral load test

^g^Documented in HIV medical chart or reported by family members upon tracing

**Table 4:** Antiretroviral therapy initiation by 90 days of HIV diagnosis, and combined antiretroviral therapy initiation and retention by 18 months of diagnosis, among clients who received CommLink and peer-delivered standard linkage services, by demographic characteristics, Manzini region, Eswatini, March 2016 – April 2020

	**Unadjusted**		**Adjusted**^**a**^			
**Characteristics**	**CommLink** **n (%)**	**SLS** **n (%)**	**CommLink** **% (95% CI)**	**SLS** **% (95% CI)**	**RR (95% CI)**	***p*-value**
**ART initiated by 90 days of diagnosis**^b^						
Sex						
Men	390 (88.8)	111 (34.8)	86.5 (85.1, 88.0)	33.7 (27.3, 41.5)	2.57 (2.08, 3.17)	< 0.0001
Women	309 (92.5)	191 (42.4)	90.5 (88.3, 92.7)	41.2 (34.7, 48.9)	2.20 (1.83, 2.65)	< 0.0001
Age group (years)						
15–24	106 (93.0)	61 (33.3)	90.9 (85.2, 97.0)	32.2 (25.7, 40.3)	2.83 (2.28, 3.51)	< 0.0001
25–34	301 (89.3)	138 (38.0)	86.9 (83.8, 90.1)	36.8 (30.3, 44.8)	2.36 (1.92, 2.90)	< 0.0001
≥ 35	292 (90.7)	103 (46.2)	88.9 (85.7, 92.3)	44.1 (34.3, 56.7)	2.02 (1.58, 2.58)	< 0.0001
Test location (CommLink ART eligibility period)^c^						
Urban (CD4 ≤ 500)	195 (78.6)	110 (43.8)	78.6 (76.5, 80.9)	43.9 (40.9, 47.0)	1.79 (1.72, 1.87)	< 0.0001
Rural (test and treat)	504 (96.0)	192 (37.1)	96.2 (94.9, 97.4)	37.1 (31.6, 43.6)	2.59 (2.20, 3.04)	< 0.0001
**ART initiated and retained by 18 months of diagnosis**^b,d^						
Sex						
Men	343 (78.1)	145 (45.5)	75.4 (66.2, 85.9)	44.5 (37.9, 52.2)	1.70 (1.39, 2.07)	< 0.0001
Women	269 (80.5)	243 (54.0)	79.8 (75.5, 84.3)	55.1 (49.4, 61.4)	1.45 (1.24, 1.69)	< 0.0001
Age group (years)						
15–24	82 (71.9)	81 (44.3)	73.3 (68.8, 78.2)	45.1 (36.9, 55.3)	1.62 (1.35, 1.95)	< 0.0001
25–34	260 (77.2)	178 (49.0)	76.9 (72.4, 81.8)	48.9 (41.6, 57.4)	1.57 (1.32, 1.88)	< 0.0001
≥ 35	270 (83.9)	129 (57.8)	84.8 (80.7, 89.1)	57.4 (47.7, 69.0)	1.48 (1.18, 1.85)	0.0007
Test location (CommLink ART eligibility period)^c^						
Urban (CD4 ≤ 500)	186 (75.0)	141 (56.2)	72.8 (66.5, 79.8)	55.4 (52.1, 59.0)	1.31 (1.13, 1.53)	0.0005
Rural (test and treat)	426 (81.1)	247 (47.7)	78.4 (76.0, 80.9)	47.0 (41.0, 53.9)	1.67 (1.45, 1.93)	< 0.0001

*SLS* peer-delivered standard linkage services, *ART* antiretroviral therapy, *RR* relative risk, *CI* confidence interval, *Tinkhundla* regional geopolitical subdivisions of Eswatini

^a^Estimated using generalized estimating equations (GEE) models with a log link (SAS 9.4) adjusting for age group, sex, urban or rural area of HIV diagnosis, and within-Tinkhundla clustering

^b^Among all CommLink (773) and SLS (769) clients

^c^SLS clients were HIV diagnosed during test and treat when all persons with HIV infection were eligible to receive ART

^d^Retained is defined as not being more than 90 days late for the last antiretroviral refill appointment

After ART initiation, proportionally more CommLink (17.3%) than SLS (14.6%) clients transferred care to at least one other facility (Table 3). At initiation or transfer facilities, fewer CommLink than SLS clients were retained on ART 24 months after initiation (84.8% vs. 88.3%), and of 566 (75.6%) CommLink and 359 (71.9%) SLS clients who had at least one documented viral load test, fewer CommLink than SLS clients were virally suppressed at their last test (93.7% vs. 95.4%) (Table 3, Fig. 4b). Of those virally suppressed at their last test, 89.7% (481/536) of CommLink and 91.3% (313/343) of SLS clients had a viral load ≤ 40 copies/mL. Of 107 facilities where clients ever received ART, 56 (52%) were in regions other than Manzini, and 49 (46%) served ≥ 1 clients of both groups including 733 (98%) CommLink and 476 (95%) SLS clients who had ever initiated ART (Fig. 1, Supplementary Table 5, Additional file 1).

In combined ART initiation and retention analyses, twice as many CommLink than SLS clients were initiated and retained on ART by 6 months of diagnosis, decreasing to 54% more CommLink than SLS clients by 18 months of diagnosis (Table 3). Across sex, age, and urban and rural subgroups, 31% to 70% more CommLink than SLS clients were initiated and retained on ART by 18 months of diagnosis (Table 4).

**Fig. 5:**
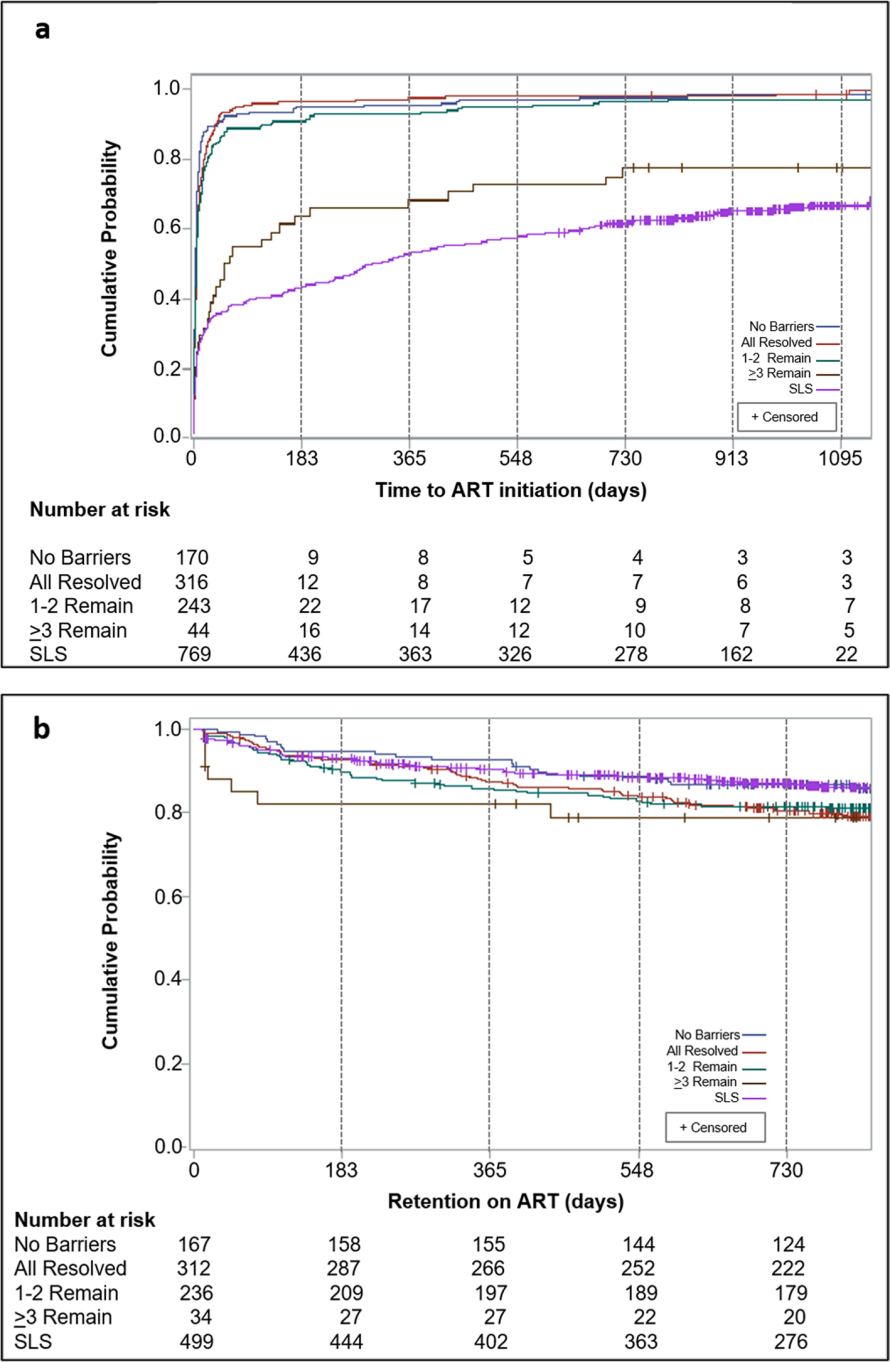
Kaplan–Meier estimates of time from HIV diagnosis in community settings in Manzini region to facility-based antiretroviral therapy initiation (**a**), and time retained on antiretroviral therapy after initiation (**b**), by peer-delivered standard linkage service and CommLink barriers-to-care subgroups, Eswatini, March 2016 – April 2020. *SLS* peer-delivered standard linkage services, *ART* antiretroviral therapy, *Resolved* barriers judged by CommLink peer counselors at the end of case management to no longer interfere with or prevent early enrollment or retention in HIV care, *Remain* barriers judged by CommLink peer counselors at the end of case management to interfere with or prevent early enrollment or retention in HIV care

During a median case-management period of 52 days (IQR 35—69), CommLink peer counselors identified ≥ 1 barriers among 603 (78%) clients, and helped resolve 896 (65%) of 1372 barriers. Counselors helped resolve 100% of barriers of 316 (41%) clients, 44% of barriers of 243 (31%) clients with 1–2 remaining unresolved barriers, and 9% of barriers of 44 (6%) clients with ≥ 3 remaining unresolved barriers (Table 5). Of clients with ≥ 3 unresolved barriers, the most frequent unresolved barriers included non-disclosure, having too many responsibilities, perceived wellness, and concerns about loss of confidentiality and stigmatization. Compared with clients with ≥ 3 unresolved barriers to care, at least 62% more CommLink clients without identified barriers or whose counselors resolved all or all but 1–2 barriers, initiated ART within 90 days of diagnosis, and at least 42% more initiated and remained on ART by 18 months of diagnosis (Table 6). Time to ART initiation curves never overlapped between SLS clients and CommLink barriers-to-care sub-groups, including CommLink clients with ≥ 3 unresolved barriers (Fig. 5a). Time retained on ART curves overlapped only between SLS clients and CommLink clients whose counselors did not identify any barriers during case management (Fig. 5b).

**Table 5:** Total identified and unresolved barriers to enrollment or retention in HIV care among CommLink clients, Manzini region, Eswatini, March 2016 – September 2018^a^

	**All Clients** **(*n* = 773)**		**All Barriers Resolved**^**b**^ **(*n* = 316)**		**1–2 Barriers Unresolved**^**c**^ **(*n* = 243)**		** ≥ 3 Unresolved Barriers**^**c**^ **(*n* = 44)**	
**Enrollment & Retention Barriers**	**Total** **Barriers**	**Unresolved** **Barriers**	**Total** **Barriers**	**Unresolved** **Barriers**	**Total** **Barriers**	**Unresolved** **Barriers**	**Total** **Barriers**	**Unresolved** **Barriers**
Total (% of total barriers)	1372	476 (35)	637	–	551	308 (56)	184	168 (91)
Median (IQR)	1 (1–3)	0 (0–1)	2 (1–3)	–	2 (1–3)	1 (1–2)	4 (3–5)	3 (3–4)
Type (% of clients)								
Non-disclosure^d^	–	123 (16)	–	–	–	100 (41)	–	23 (52)
Too busy^e^	241 (31)	51 (7)	129 (41)	–	82 (33)	28 (12)	30 (68)	23 (52)
Perceived wellness^f^	95 (12)	24 (3)	43 (14)	–	31 (13)	4 (2)	21 (48)	20 (45)
Concerned about stigma^g^	134 (17)	36 (5)	63 (20)	–	52 (21)	18 (7)	19 (43)	18 (41)
Fears response from or loss of partner^h^	101 (13)	30 (4)	49 (16)	–	39 (16)	18 (7)	13 (30)	12 (27)
Excessive alcohol use	136 (18)	73 (9)	58 (18)	–	65 (27)	61 (25)	13 (30)	12 (27)
Denies having HIV^i^	60 (8)	13 (2)	30 (9)	–	19 (8)	3 (1)	11 (25)	10 (23)
Costs are too high^j^	81 (10)	23 (3)	47 (15)	–	25 (10)	14 (6)	9 (20)	9 (20)
ART has side effects or is ineffective^k^	81 (10)	14 (2)	52 (16)	–	19 (8)	5 (2)	10 (23)	9 (20)
Believes in traditional medicine	18 (2)	8 (1)	6 (2)	–	6 (2)	2 (1)	6 (14)	6 (14)
Believes prayer can prevent or cure AIDS	16 (2)	6 (1)	7 (2)	–	4 (2)	1 (0)	5 (11)	5 (11)
HIV-care providers are disrespectful	44 (6)	5 (1)	28 (9)	–	11 (5)	1 (0)	5 (11)	4 (9)
Quality of HIV care is poor^l^	40 (5)	4 (1)	27 (9)	–	9 (4)	0	4 (9)	4 (9)
Other	202 (26)	66 (9)	98 (31)	–	89 (37)	53 (22)	15 (34)	13 (30)

*IQR* interquartile range, *ART* antiretroviral therapy, *AIDS* acquired immunodeficiency syndrome

^a^Barriers were routinely assessed and recorded on a standard form throughout the CommLink case management period (median 52 days, IQR 35—69)

^b^Judged by peer counselors at the end of case management to no longer interfere with or prevent early enrollment or retention in HIV care

^c^Judged by peer counselors at the end of case management to interfere with or prevent early enrollment or retention in HIV care

^d^Did not disclose HIV status to any sexual partners or family members during case management

^e^Too busy with work, family, or other responsibilities to enroll or remain in HIV care

^f^Does not believe enrolling in HIV care and ART is needed because of perceived good health and wellbeing

^g^Fears loss of confidentiality and stigma when visiting healthcare facilities

^h^Fears lack of support, violence, or separation from spouse or sexual partner

^i^Believes that the HIV test results were wrong and denies having HIV

^j^Believes transportation costs or costs from loss of work are too high

^k^Believes ART has severe side effects or is ineffective

^l^Believes that the quality of HIV care is poor and does not trust healthcare providers

**Table 6:** Antiretroviral therapy initiation by 90 days of HIV diagnosis, and combined antiretroviral therapy initiation and retention by 18 months of diagnosis, by barriers-to-care subgroups, CommLink clients**,** Manzini region, Eswatini, March 2016 – April 2020

	**Unadjusted**		**Adjusted**^**a**^		
**Characteristics**	**Clients**	**n (%)**	**% (95% CI)**	**RR (95% CI)**	***p*-value**
ART Initiated by 90 days of diagnosis^b^					
≥ 3 unresolved barriers	44	24 (54.6)	53.8 (35.2, 82.2)	Referent	
1–2 unresolved barriers	243	216 (88.9)	87.3 (85.5, 89.0)	1.62 (1.05, 2.51)	0.0296
All barriers resolved	316	301 (95.3)	89.8 (87.8, 92.0)	1.67 (1.09, 2.55)	0.0177
No barriers	170	158 (92.9)	90.6 (88.2, 93.0)	1.69 (1.08, 2.63)	0.0212
ART initiated and retained by 18 months of diagnosis^b,c^					
≥ 3 unresolved barriers^d^	44	24 (54.6)	53.2 (45.1, 62.9)	Referent	
1–2 unresolved barriers	243	188 (77.4)	75.4 (70.6, 80.4)	1.42 (1.19, 1.68)	< 0.0001
All barriers resolved	316	255 (80.7)	76.6 (73.7, 79.5)	1.44 (1.25, 1.66)	< 0.0001
No barriers	170	145 (85.3)	81.8 (78.1, 85.6)	1.54 (1.30, 1.81)	< 0.0001

*SLS* peer-delivered standard linkage services, *ART* antiretroviral therapy, *RR* relative risk, *CI* confidence interval, *Tinkhundla* regional geopolitical subdivisions of Eswatini

^a^Estimated using generalized estimating equations (GEE) models with a log link (SAS 9.4) adjusting for age group (initiated and retained by 18 months of diagnosis only), sex, urban or rural area of HIV diagnosis, and within-Tinkhundla clustering (model did not converge with age-group variable included for ART initiated by 90 days of diagnosis)

^b^Among all CommLink clients (*n* = 773)

^c^Retained is defined as not being more than 90 days late for the last antiretroviral refill appointment

^d^Clients initiated and retained on ART by 18 months of diagnosis (*n* = 24) are not all the same clients as those who initiated ART by 90 days of diagnosis (*n* = 24)

Few CommLink (2.8%) and SLS (1.8%) clients were documented by medical record abstraction (*n* = 22) or reported by family members upon tracing (*n* = 14) to have died. Of 504 clients not known to have died and not initiated and retained on ART, 501 (99%) had been traced by facility or PSI program staff. Of these, 79 (CommLink 37, SLS 42) clients were contacted, confirmed not to be on ART, and referred for HIV care (care status unknown); 28 were reported by family members to have moved to South Africa or Mozambique; and 394 remained lost to follow-up.

## Discussion

Consistent with more than 15 studies in sub-Saharan Africa suggesting that half or fewer community-diagnosed PLHIV provided a referral as the only linkage service enroll early in HIV care, few SLS clients who received a single peer-delivered counseling and referral session with telephone follow-up enrolled early in HIV care, exceptionally few initiated ART within 7 days of diagnosis, and many delayed their enrollment in care for more than one year after diagnosis [12–21]. Notably, ART initiation within 90 days of diagnosis among SLS clients (38%) was similar with enrollment in pre-ART care among clients diagnosed in community settings in Eswatini during 2011–2013 (17%-34%), suggesting that community-diagnosed PLHIV remain highly vulnerable to barriers to care in the test and treat era [14, 15]. Although ART initiation among SLS clients steadily increased over time, 54% more CommLink clients were initiated and retained on ART by 18 months of diagnosis. Encouragingly, CommLink peer counselors helped resolve a majority of identified barriers for most clients, and similar high proportions of CommLink clients without any identified barriers as those whose counselors helped resolve all or all but 1–2 barriers were initiated on ART within 90 days of diagnosis and were retained on ART by 18 months of diagnosis.

ART initiation within 90 days of diagnosis among CommLink clients during test and treat (96%) is consistent with findings from a similar peer-delivered, linkage case management program in Tanzania that enrolled in care 96% of 1,900 clients during CD4 ≤ 500 and test and treat ART-eligibility periods, and with two community-based studies in Uganda and South Africa that provided follow-up services and achieved > 90% early enrollment in care [13, 26]. Findings from our study comparing two peer-delivered programs suggest that the mixed findings of a systematic review of peer-delivered interventions to improve early ART initiation and retention might be attributed to variations in the content and duration of linkage and retention services [25]. Services that are limited to a single face-to-face counseling session with telephone follow-up are likely insufficient to help many clients with important barriers, even if these services are delivered by HIV-positive peer counselors [4–11]. Although our study design did not permit evaluating the efficacy of component services, we believe all CDC and WHO recommended services are integral to early ART initiation and retention by helping many clients avoid, mitigate, or resolve barriers to care [3, 23, 24].

Central to these services was a dedicated HIV-positive, ART-adherent case manager who had time to build trusting relationships essential for understanding individual circumstances and delivering effective client-centered services. Peer counselors routinely disclosed their HIV status at the first session, showing their own treatment cards and time on ART to normalize the importance and benefit of immediate and sustained treatment. Peers used their training and personal experiences with HIV to help clients cope with their diagnosis, plan when and how to tell sex partners and family members when appropriate and safe, enroll in care and initiate ART as soon as possible, troubleshoot treatment side-effects and negative healthcare experiences, and when needed, help clients transfer care to a more suitable facility [29]. Often called by their clients, CommLink peer counselors provided real-time support when questions, adverse experiences, and fears emerged in the two–three months following diagnosis.

Retention on ART two years after initiation among CommLink (85%) and SLS (88%) clients was similar with two near-contemporary patient cohorts in Eswatini (81%-85%), and was greater than most patient cohorts in sub-Saharan Africa in studies that addressed (77%-80%) and did not address (71%-74%) undocumented self-transfer and mortality [30–35]. Although a small but significantly higher proportion of ART-initiated SLS than CommLink clients remained on ART and achieved viral load suppression, this finding may be attributed to differences in unresolved barriers to care at ART initiation. The 64% of SLS clients who initiated ART enrolled on their own without treatment-navigation services, and for most, after having coped with their diagnosis and potential barriers for six months or longer. In contrast, nearly all (97%) CommLink clients initiated ART soon after diagnosis, including some of those with ≥ 3 unresolved barriers to care who were unsurprisingly at higher risk for discontinuing ART when case-management services ended. Notably, Kaplan–Meier ART retention curves overlapped between SLS clients and CommLink clients who did not have any identified barriers.

Although CommLink achieved near universal early ART initiation during test and treat, our findings underscore the challenges of achieving ambitious targets (> 90%) for early ART initiation *and* retention for PLHIV who were likely not seeking medical care at the time of their diagnosis. CommLink peer counselors did not resolve all barriers for all clients, and they resolved few barriers for an important minority of clients who were most likely to delay ART initiation or default early from care. Although peer counselors routinely introduced and transitioned their clients for ongoing support with facility-based adherence counselors, the nature of retention services provided after CommLink services ended is unknown. The diversity of unresolved barriers among CommLink clients at highest risk for not initiating or discontinuing ART is consistent with many studies suggesting that reasons for not engaging in care are complex, and that multidisciplinary teams (e.g., peer counselors, social workers, psychologists, medical staff) might be needed to identify solutions for clients in the most challenging of circumstances [4–11].

Our findings suggest that routinely measuring resolved and unresolved barriers to care might be used to strengthen linkage and retention services by identifying high-risk clients for differentiated service delivery, particularly during the critical first six months of treatment when default rates are highest [32, 34–36]. We measured barriers from diagnosis through the end of case management as part of routine client-centered counseling. Research is needed to evaluate whether measurement of unresolved barriers might be combined with other measures (e.g., poverty) to develop a valid and reliable index to identify high-risk clients for differentiated services, and to evaluate whether these services are effective at reducing early losses to the HIV-care cascade [36].

Findings from our study are subject to at least four limitations. First, because the study was not a randomized controlled trial, some differences in outcomes could be attributed to unmeasured confounding, such as differential healthcare-access barriers (e.g., distance from residence to facilities). We attempted to minimize this limitation by comparing approximate equal samples of clients diagnosed in urban (presumably shorter distances) and rural (presumably longer distances) areas. Although facility factors (e.g., quality of care) within these strata could remain an important confounder, nearly all CommLink and SLS ART-initiated clients were served by the same facilities, and ART retention was similarly high between CommLink, SLS, and other Eswatini patient cohorts [30, 31]. Second, the frequency and distribution of barriers to care among SLS clients was not measured and is unknown. Although we cannot rule out that SLS clients at diagnosis had more barriers to care (accounting for lower ART initiation rates), proportionally more CommLink than SLS clients were men who presumably have more barriers than women because they consistently underutilize HIV care [1, 3, 24]. Additionally, nearly one-third of CommLink clients were diagnosed before test and treat, and thus faced a substantial policy barrier to early ART that was not experienced by SLS clients. Third, our methods are subject to omissions and errors in clinical records and identifying enrolled clients. We attempted to minimize potential bias with comprehensive overlapping search procedures, verifying transfers to any facility in Eswatini, and enabling defaulter tracing at healthcare facilities and by PSI. Despite these procedures, our ART retention estimates likely remain underestimated for both cohorts because of undocumented mortality or transfer of care [35]. Finally, CommLink peer counselors determined on their own when identified barriers were resolved, and the validity of their judgement for each barrier is unknown. Resolved barriers are restricted to the average two-month case management period only and are not assumed to be extinguished.

## Conclusions

Compared with peer-delivered standard referral, counseling, and telephone-based linkage services, twice as many clients who received peer-delivered, linkage case management services over an average two-month period enrolled in HIV care and initiated ART by 90 days of diagnosis. Although enrollment in HIV care steadily increased over time among clients who received standard services, 54% more clients who received CommLink services were initiated and retained on ART 18 months after diagnosis. To improve early ART initiation and retention among PLHIV diagnosed in community settings, HIV prevention and treatment programs should consider implementing a package of peer-delivered linkage case management services [3, 23, 24, 27]. Clients with multiple unresolved barriers to care measured as part of that package should be triaged for differentiated linkage and retention services to help reduce losses to the HIV-care cascade. In 2019, the Eswatini Ministry of Health adopted linkage case management as standard of care for clients diagnosed in facility and community settings [37]. In 2021, the U.S. President’s Emergency Plan for AIDS Relief supported an expanded 6-month linkage case management model in Eswatini to help address unresolved barriers to care and improve ART retention [38].

## Supplementary Information

**Additional file 1: Table S1.** CommLink and SLS urban and rural sample sizes. **Table S2.** CommLink barriers form. **Table S3.** Study data abstraction form. **Table S4.** SAS-based Search Algorithm. **Table S5.** Study referral and ART facilities

## Data Availability

Datasets analyzed for this study are governed by the Eswatini Health Research Review Board (EHRRB) and are not publicly available to protect patient confidentiality. Datasets may be made available with EHRRB approval. To request permission to access study datasets, interested persons should contact the corresponding author who will facilitate requests with EHRRB.
