# Supplementary material for: Antiretroviral therapy initiation and retention among clients who received peer-delivered linkage case management and standard linkage services, Eswatini, 2016–2020: retrospective comparative cohort study
Source: Arch Public Health. 2022 Mar 9;80:74. doi: 10.1186/s13690-022-00810-9 (PMC8905856; doi:10.1186/s13690-022-00810-9)
Supplement: Supplementary file 1 — Additional file 1: Table S1. CommLink and SLS urban and rural sample sizes. Table S2. CommLink barriers form. Table S3. Study data abstraction form. Table S4. SAS-based Search Algorithm. Table S5. Study referral and ART facilities [file 13690_2022_810_MOESM1_ESM.docx]

**Table S1** CommLink and SLS urban and rural sample sizes

| Characteristics | CommLink | SLS |
| --- | --- | --- |
| Urban |  |  |
| HIV-testing period^a^ | Mar 2016 – Sep 2016 | Oct 2016 – Jan 2018 |
| ART eligibility^b^ | CD4<500 | Test and Treat |
| Eligible sample (n)^c^ | 248^d^ | 251 |
| Tinkhundla (eligible sample)^e^ |  |  |
| Manzini - North | 113 | 165 |
| Manzini - South | 135 | 86 |
| Rural |  |  |
| HIV-testing period^a^ | Oct 2016 – Mar 2018 | Oct 2016 – Feb 2018 |
| ART eligibility^b^ | Test and Treat | Test and Treat |
| Eligible sample (n)^c^ | 525 | 518 |
| Tinkhundla (eligible sample)^e^ |  |  |
| Kukhanyeni | 0 | 32 |
| Lamgabhi | 1 | 38 |
| Lobamba Lomdzala | 0 | 114 |
| Ludzeludze | 147 | 113 |
| Mafutseni | 13 | 19 |
| Mahlangatsha | 0 | 31 |
| Mangcongco | 0 | 20 |
| Mhlambanyatsi | 0 | 15 |
| Mkhiweni | 0 | 49 |
| Mtfongwaneni | 0 | 31 |
| Ngwempisi | 51 | 14 |
| Nhlambeni | 21 | 37 |
| Ntondozi | 292 | 5 |

*SLS* peer-delivered standard linkage services, *ART* antiretroviral therapy, *Tinkhundla* geopolitical subdivisions of Manzini region, Eswatini ^a^Excluding the provision of index testing services for partners and family members, CommLink and SLS teams did not operate in the same urban and rural Tinkhundla during the same months

^b^National guidelines recommending ART based on CD4 count were expanded during the study period, resulting in the following two ART-eligibility periods: 1 March 2016 to 30 September 2016 (CD4 count <500/μL) and 1 October 2016 to 31 March 2018 (any CD4 count, test and treat)

^c^Clients aged >15 years who tested HIV-positive, had not received HIV care in the prior 90 days, consented for follow-up services, and were referred for HIV care in any healthcare facility in Manzini region or in regional border zones

^d^7 clients were partners or family members of CommLink clients who tested HIV positive during outreach testing in rural Tinkhundla during test and treat

^e^During 1 March 2016 to 31 March 2018, PSI provided community-based testing services in all but one (Kwaluseni) of the 16 Manzini Tinkhundla; Kwaluseni (Matsapha) was served by a different community-based testing provider; after the CommLink program in 2018, two additional Tinkhundla were designated in Manzini (Phondo, Nkomiyahlaba)

**Table S2** CommLink barriers form

Part A. Enrollment Barriers

| **Enrollment Barrier** | **Barrier Summary** | **Barrier Present** | | **Barrier Resolved** | |
| --- | --- | --- | --- | --- | --- |
|  |  | YES | NO | YES | NO |
| 1. Feeling well; no need to go to HIV clinic now. | *Client does not believe he/she needs to go to the HIV clinic because of perceived good health.* |  |  |  |  |
| 2. No time; too busy with work or other responsibilities. | *Client believes he/she does not have the time to go to CTCs.* |  |  |  |  |
| 3. Transportation costs or costs from loss of work are too high. | *Client believes costs (e.g., transportation, loss of work, family) are barriers to care.* |  |  |  |  |
| 4. Health-care providers at CTCs are disrespectful. | *Client believes he/she will be disrespected or treated poorly at the CTC.* |  |  |  |  |
| 5. ART does not work; ART has bad side effects. | *Client believes that he/she will remain healthy without going to the CTC.* |  |  |  |  |
| 6. Does not trust health-care providers; poor quality of care at CTCs. | *Client does not trust health care providers or believes that the quality of care is poor.* |  |  |  |  |
| 7. Loss of confidentiality and stigma. | *Client is does not want others to know and fears stigmatization if identified at the CTC.* |  |  |  |  |
| 8. Fears lack of support, violence, or separation from spouse or partner. | *Client fears disclosing, and lack of support, violence, or loss of spouse or regular partner.* |  |  |  |  |
| 9. Denies being HIV infected. | *Client believes he/she is not infected with HIV.* |  |  |  |  |
| 10. Believes in traditional medicine. | *Client believes that traditional medicine cures HIV or AIDS.* |  |  |  |  |
| 11. Has strong religious beliefs. | *Client believes that prayer will cure HIV or AIDS.* |  |  |  |  |
| 12. Other? | *Other barrier (summarize):* |  |  |  |  |
| 13. Is using alcohol and/or other substances. | *Client has been drinking too much and/or using other drugs.* |  |  |  |  |

Part B. Retention Barriers

| **Retention Barriers** | **Barrier Summary** | **Barrier Present** | | **Barrier Resolved** | |
| --- | --- | --- | --- | --- | --- |
|  |  | YES | NO | YES | NO |
| 1. Feeling well; no need to return to HIV clinic now. | *Client does not believe he/she needs to return to the HIV clinic because of perceived good health.* |  |  |  |  |
| 2. No time; too busy with work or other responsibilities. | *Client believes he/she does not have the time to make all appointments at CTC.* |  |  |  |  |
| 3. Transportation costs or costs from loss of work are too high. | *Client believes costs or responsibilities (e.g., transportation, work) are retention barriers.* |  |  |  |  |
| 4. Health-care providers at CTCs are disrespectful. | *Client has been treated poorly at the CTC or seen others treated poorly at the CTC.* |  |  |  |  |
| 5. ART does not work; ART has bad side effects. | *Client believes that ART has bad side effects or does not work.* |  |  |  |  |
| 6. Does not trust health-care providers; poor quality of care at CTCs. | *Client does not trust health care providers or believes that the quality of care is poor.* |  |  |  |  |
| 7. Loss of confidentiality and stigma. | *Client does not want others to know and fears stigmatization if identified at the CTC.* |  |  |  |  |
| 8. Fears separation from spouse or partner. | *Client fears disclosing and loss of spouse or main partner.* |  |  |  |  |
| 9. Believes in traditional medicine. | *Client believes that traditional medicine cures HIV or AIDS.* |  |  |  |  |
| 10. Has strong religious beliefs. | *Client believes that prayer will cure HIV or AIDS.* |  |  |  |  |
| 11. Other? | *Other barrier (summarize):* |  |  |  |  |
| 12. Is using alcohol and/or other substances. | *Client has been drinking too much and/or using other drugs.* |  |  |  |  |

**Table S3** Study data abstraction form

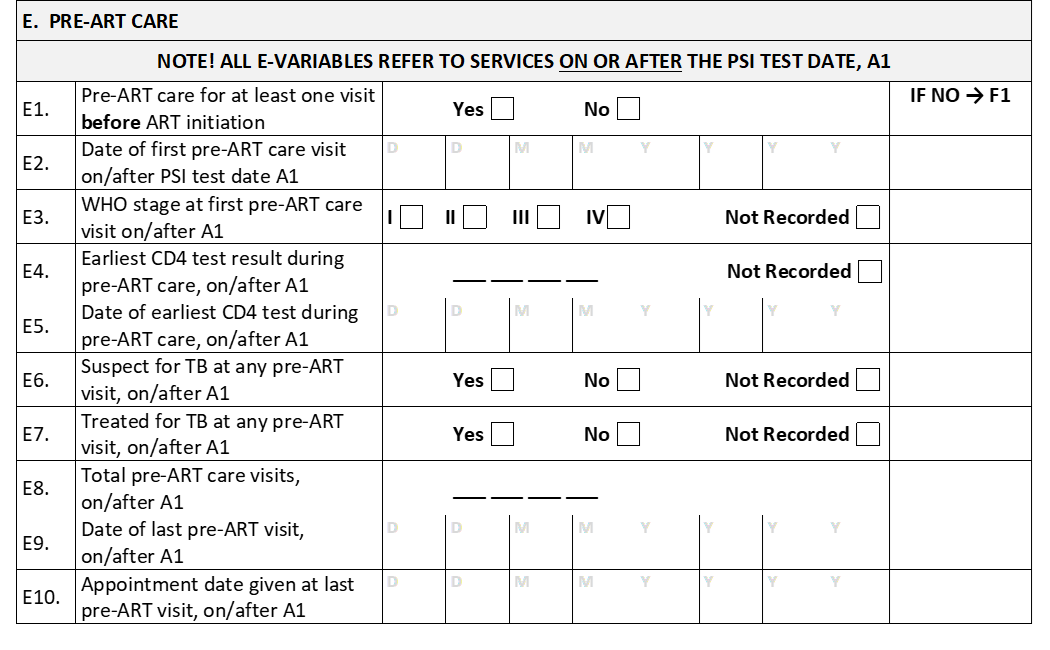

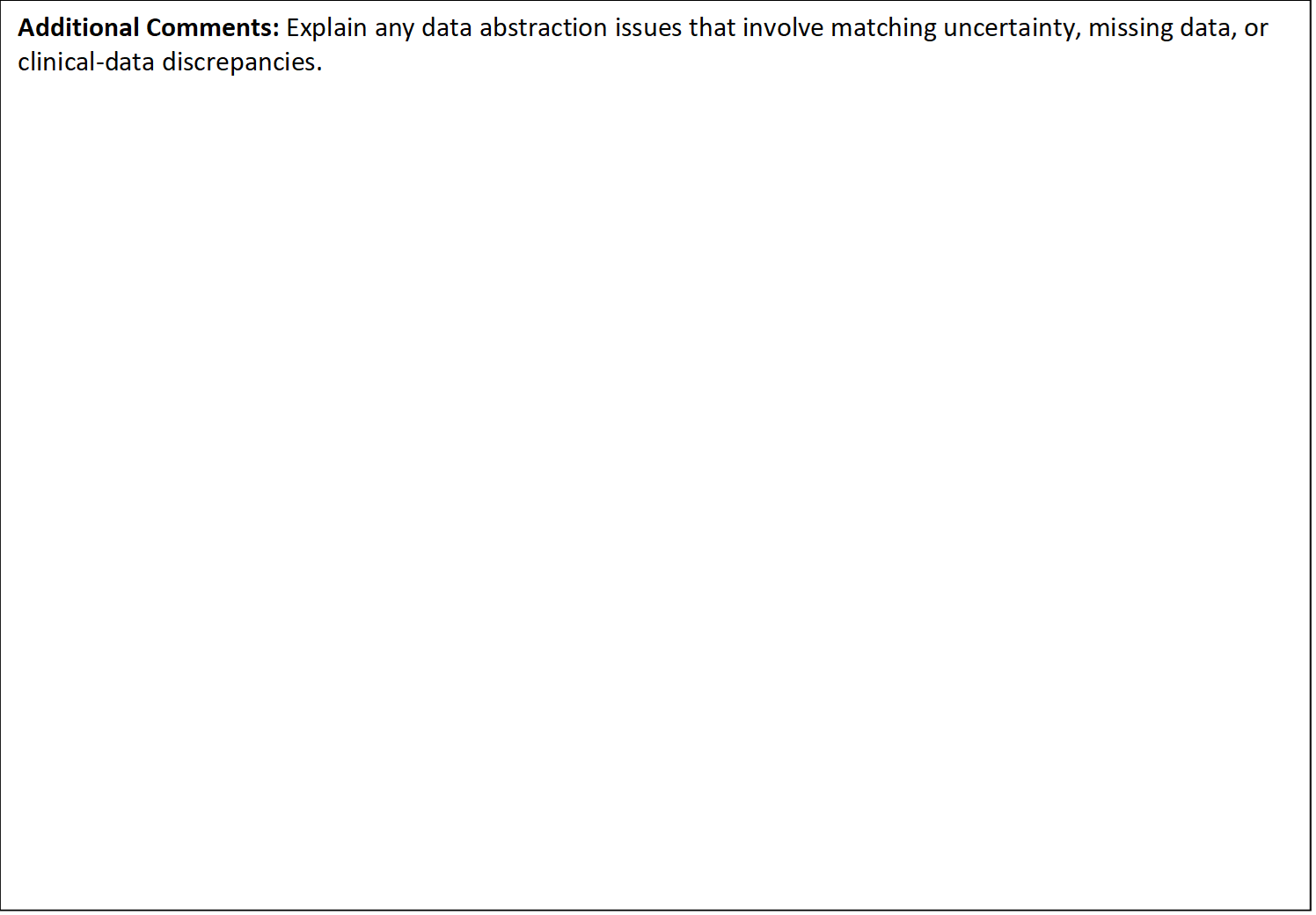


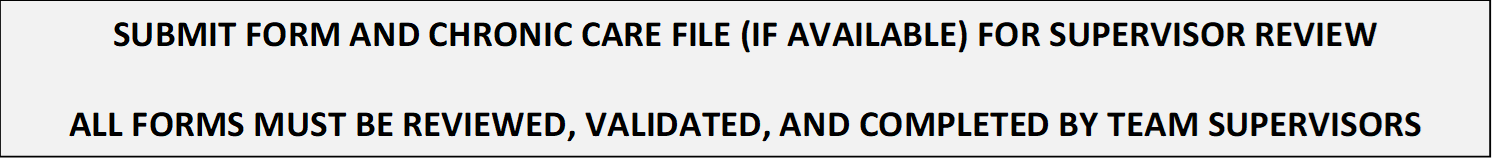


I confirm that all the Supervisor-indicated data recorded on this form has been validated and is identical with that recorded in the Chronic Care File or other medical records used to abstract data (if applicable):

Signature of Supervisor: _________________________________ Date: ___ ___ / ___ ___ / ___ ___

Supervisor ID Code: ___ ___ ___

**Table S4** SAS-based Search Algorithm

The following variables available in the study cohort dataset were used to generate potential matches when available in the National ART database (variables marked with an asterisk were not consistently available in both sources):

- - Full Name
  - First Name
  - Surname
  - Gender
  - Date of Birth Year
  - Age*
  - Date of Birth*
  - Phone Number*
  - Next of Kin Name*
  - Next of Kin Phone Number*
  - ART number*

A unique record ID was created for each record in each database searched. A linking variable was created (linkvar = 1) in each database to link every record in the National ART database to every record in the study cohort dataset for comparison. This step resulted in a very large file with the total number of records equal to *a***b* where *a* is the number of records in the study cohort dataset, and *b* is the number of records in the National ART database.

The SAS function COMPLEV was used to compare character string values. COMPLEV returns the Levenshtein edit distance between string-1 and string-2. The Levenshtein edit distance is the number of insertions, deletions, or replacements of single characters that are required to convert one string to the other. Reference: <https://documentation.sas.com/?docsetId=lefunctionsref&docsetTarget=n0l41pdemybegln1oetsh4cctdap.htm&docsetVersion=9.4&locale=en>

The following comparisons were performed, when available, between the variables in the study cohort dataset and the National ART database to search for potential matches:

- - Full Name Comparison: Compare “Full Name” using the SAS COMPLEV function
  - First Name Comparison: Compare “First Name” using the SAS COMPLEV function
  - Last Name Comparison: Compare “Last Name” using the SAS COMPLEV function
  - Gender Comparison: If Gender matches, then 0. If Gender does not match, then 1.
  - Date of Birth Year Comparison: Compare the date of birth year string using the SAS COMPLEV function
  - Date of Birth Year Difference: Absolute value of the difference between the values of date of birth year
  - Date of Birth Difference: The absolute value of difference in days between the value of date of birth
  - Age Difference: The absolute value of the difference between the value of age
  - Phone Number Comparison: Compare “Phone Number” using the SAS COMPLEV function
  - ART Number Comparison: Compare ART Number using the SAS COMPLEV function
  - Combined Score: The sum of the COMPLEV function scores from comparing Full Name, Gender, and Date of Birth Year

If there were a minimum of two exact matches on any combination of the comparisons (above), then the record was included in the set of potential matches if at least one, but no more than two, of the calculations were within the following thresholds:

| Comparison Calculation | Threshold |
| --- | --- |
| Full Name Comparison | COMPLEV <= 5 |
| First Name Comparison | COMPLEV <= 5 |
| Last Name Comparison | COMPLEV <= 5 |
| Gender Comparison | <= 1 |
| Date of Birth Year String Comparison | COMPLEV <= 2 |
| Date of Birth Year Difference | <= 5 |
| Date of Birth Difference | <= 1825 |
| Age Difference | <= 5 |
| Phone Number Comparison | COMPLEV <= 2 |
| ART Number Comparison | COMPLEV <= 1 |
| Combined Score | <= 14 |

For records with no potential matches that met the above criteria, then the minimum combined score for each set of potential matches for each HIV testing number was used to include the record in the set potential matches.

| Comparison Calculation | Threshold |
| --- | --- |
| First Name Comparison | COMPLEV <= 5 |
| Last Name Comparison | COMPLEV <= 5 |
| Date of Birth Year String Comparison | COMPLEV <= 1 |
| Date of Birth Year Difference | <= 5 |

To reduce the number of fuzzy matches in the final output, for records with more than 10 potential matches, records were excluded from the set of potential matches if there was not a minimum of three exact matches on any combination of the Comparison Calculations, and one or more values were outside of the following thresholds:

Sorting: The final output dataset of potential matches was then sorted in ascending order by the following variables to rank the potential matches from most probable to least probable.

- - Gender Comparison
  - Date of Birth Year Comparison
  - Date of Birth Year Difference
  - Full Name Comparison
  - Last Name Comparison
  - First Name Comparison
  - Date of Birth Comparison
  - Age Difference
  - ART Number Comparison
  - Phone Number Comparison
  - Combined Score

The SAS-based search algorithm was validated on December 11, 2019 using 579 completed abstractions of clients who were located and matched to HIV clinical records at healthcare facilities without using the national ART database. The algorithm was applied to a 25% random sample of these cases (n=145) to determine the percentage of facilities at which clients were matched that were identified by the algorithm. The algorithm produced an average list of 6 potential matches per client. For 133 of the 145 cases (92%), at least one of the facilities in the list of potential matches produced by algorithm matched the facility where clients were located and matched to clinical records. For 99 of the 133 cases (74%), the matching record was the highest ranked (first) potential match produced by the algorithm.

**Table S5** Study referral and ART facilities

|  |  | Referred for HIV Care^a^ | | | Ever Received ART^b^ | | |
| --- | --- | --- | --- | --- | --- | --- | --- |
| Healthcare Facility | Region | CommLink | SLS | Total | CommLink | SLS | Total |
| AHF MATSAPHA | Manzini | 30 | 98 | 128 | 27 | 63 | 90 |
| AHF MBABANE | Hhohho | 0 | 0 | 0 | 7 | 0 | 7 |
| AHF NHLANGANO | Shiselweni | 0 | 0 | 0 | 1 | 3 | 4 |
| BAYLOR CENTER OF EXCELLENCE CLINIC | Hhohho | 1 | 0 | 1 | 4 | 0 | 4 |
| BHAHWINI CLINIC | Manzini | 2 | 0 | 2 | 1 | 0 | 1 |
| BHALEKANE CORRECTIONAL SERVICES CLINIC | Hhohho | 0 | 0 | 0 | 1 | 0 | 1 |
| BHALEKANE NAZARENE CLINIC | Hhohho | 0 | 0 | 0 | 0 | 0 | 0 |
| BHEKINKOSI NAZARENE CLINIC | Manzini | 0 | 5 | 5 | 0 | 2 | 2 |
| BHOLI CLINIC | Lubombo | 0 | 0 | 0 | 1 | 2 | 3 |
| BHUDLA CLINIC | Manzini | 0 | 1 | 1 | 0 | 0 | 0 |
| BULANDZENI CLINIC | Hhohho | 0 | 0 | 0 | 0 | 1 | 1 |
| BULUNGA NAZARENE CLINIC | Manzini | 0 | 0 | 0 | 0 | 1 | 1 |
| CABRINI HEALTH CARE | Lubombo | 0 | 0 | 0 | 0 | 0 | 0 |
| CANA ALLIANCE CLINIC | Manzini | 3 | 0 | 3 | 3 | 1 | 4 |
| DVOKOLWAKO HEALTH CENTRE | Hhohho | 1 | 10 | 11 | 2 | 5 | 7 |
| DWALILE CLINIC | Manzini | 0 | 1 | 1 | 0 | 1 | 1 |
| EBENEZER CLINIC | Lubombo | 0 | 0 | 0 | 0 | 1 | 1 |
| EKUTFOKOMENI CLINIC | Lubombo | 0 | 0 | 0 | 0 | 1 | 1 |
| EMKHUZWENI HEALTH CENTRE | Hhohho | 0 | 0 | 0 | 0 | 0 | 0 |
| EZULWINI SATELITE CLINIC | Hhohho | 1 | 0 | 1 | 2 | 3 | 5 |
| FLAS MANZINI | Manzini | 10 | 9 | 19 | 10 | 5 | 15 |
| GEBENI CLINIC | Manzini | 4 | 0 | 4 | 4 | 1 | 5 |
| GEGE CLINIC | Shiselweni | 0 | 0 | 0 | 0 | 1 | 1 |
| GILGAL CLINIC | Lubombo | 0 | 0 | 0 | 1 | 1 | 2 |
| GOOD SHEPHARD HOSPITAL | Lubombo | 0 | 0 | 0 | 1 | 2 | 3 |
| HEREFORDS CLINIC | Hhohho | 0 | 0 | 0 | 0 | 1 | 1 |
| HHUKWINI CLINIC | Hhohho | 0 | 0 | 0 | 1 | 2 | 3 |
| HLANE CLINIC | Lubombo | 0 | 0 | 0 | 1 | 0 | 1 |
| HLATHIKHULU GOVERNMENT HOSPITAL | Shiselweni | 0 | 0 | 0 | 1 | 0 | 1 |
| HLATHIKHULU PHU | Shiselweni | 0 | 0 | 0 | 0 | 1 | 1 |
| HLUTI CLINIC | Shiselweni | 0 | 0 | 0 | 0 | 0 | 0 |
| HORO CLINIC | Hhohho | 0 | 0 | 0 | 0 | 0 | 0 |
| HOSPICE AT HOME CLINIC | Manzini | 2 | 0 | 2 | 0 | 0 | 0 |
| KA MFISHANE CLINIC | Shiselweni | 0 | 0 | 0 | 0 | 1 | 1 |
| KA PHUNGA CLINIC | Shiselweni | 1 | 0 | 1 | 0 | 0 | 0 |
| KA PHUNGA NAZARENE CLINIC | Shiselweni | 0 | 0 | 0 | 0 | 0 | 0 |
| KA ZONDWAKO CLINIC | Manzini | 3 | 7 | 10 | 2 | 6 | 8 |
| KHUPHUKA CLINIC | Manzini | 0 | 4 | 4 | 1 | 3 | 4 |
| KHWEZI CLINIC | Lubombo | 0 | 0 | 0 | 0 | 1 | 1 |
| KING SOBHUZA II PUBLIC HEALTH UNIT | Manzini | 1 | 11 | 12 | 4 | 10 | 14 |
| LAMVELASE CLINIC (ZOMBODZE) | Manzini | 7 | 30 | 37 | 6 | 6 | 12 |
| LAMVELASE HELP CENTRE | Manzini | 254 | 197 | 451 | 250 | 139 | 389 |
| LOBAMBA CLINIC | Hhohho | 8 | 27 | 35 | 10 | 14 | 24 |
| LOMAHASHA CLINIC | Lubombo | 1 | 0 | 1 | 0 | 2 | 2 |
| LUBOMBO REFERRAL HOSPITAL | Lubombo | 0 | 0 | 0 | 1 | 1 | 2 |
| LUBULI CLINIC | Lubombo | 0 | 0 | 0 | 0 | 1 | 1 |
| LULAMA CLINIC | Manzini | 0 | 1 | 1 | 1 | 0 | 1 |
| LUSHIKISHINI CLINIC | Manzini | 5 | 1 | 6 | 7 | 0 | 7 |
| LUYENGO CLINIC | Manzini | 117 | 95 | 212 | 121 | 40 | 161 |
| MAFUTSENI NAZARENE CLINIC | Manzini | 4 | 7 | 11 | 6 | 3 | 9 |
| MAGUBHELENI CLINIC | Shiselweni | 1 | 2 | 3 | 1 | 1 | 2 |
| MAGUGA CLINIC | Hhohho | 0 | 0 | 0 | 0 | 0 | 0 |
| MAHLANDLE CLINIC | Shiselweni | 0 | 0 | 0 | 0 | 0 | 0 |
| MAHLANGATSHA CLINIC | Manzini | 1 | 3 | 4 | 3 | 2 | 5 |
| MAHWALALA RED CROSS CLINIC | Hhohho | 0 | 0 | 0 | 1 | 3 | 4 |
| MALINDZA REFUGEE CLINIC | Lubombo | 0 | 0 | 0 | 1 | 0 | 1 |
| MANGCONGCO CLINIC | Manzini | 4 | 2 | 6 | 3 | 4 | 7 |
| MANGWENI CLINIC | Hhohho | 0 | 0 | 0 | 0 | 1 | 1 |
| MANKAYANE GOVERNMENT HOSPITAL | Manzini | 28 | 24 | 52 | 23 | 9 | 32 |
| MANYEVENI NAZARENE CLINIC | Lubombo | 0 | 0 | 0 | 0 | 1 | 1 |
| MANZINI CITY COUNCIL CLINIC (FILTER) | Manzini | 0 | 3 | 3 | 1 | 5 | 6 |
| MANZINI CLINIC | Manzini | 0 | 0 | 0 | 0 | 2 | 2 |
| MASHOBENI CLINIC | Shiselweni | 0 | 0 | 0 | 0 | 0 | 0 |
| MATHANGENI CHURCH OF CHRIST CLINIC | Manzini | 0 | 1 | 1 | 1 | 2 | 3 |
| MATSANJENI HEALTH CENTRE | Shiselweni | 0 | 1 | 1 | 0 | 2 | 2 |
| MATSAPHA CENTRAL PRISON CORRECTIONAL CLINIC | Manzini | 0 | 0 | 0 | 1 | 0 | 1 |
| MATSAPHA HEALTH CARE CENTRE | Manzini | 0 | 0 | 0 | 1 | 0 | 1 |
| MAWELAWELA CLINIC | Manzini | 0 | 0 | 0 | 0 | 3 | 3 |
| MBABANE CLINIC | Hhohho | 0 | 0 | 0 | 0 | 0 | 0 |
| MBABANE GOVERNMENT HOSPITAL | Hhohho | 0 | 0 | 0 | 4 | 2 | 6 |
| MBABANE PUBLIC HEALTH UNIT | Hhohho | 0 | 0 | 0 | 0 | 2 | 2 |
| MBIKWAKHE CLINIC | Manzini | 20 | 30 | 50 | 28 | 9 | 37 |
| MHLAMBANYATSI CLINIC | Manzini | 0 | 2 | 2 | 0 | 2 | 2 |
| MHLUME CLINIC | Lubombo | 0 | 0 | 0 | 1 | 0 | 1 |
| MKHAYA CLINIC | Manzini | 0 | 1 | 1 | 0 | 0 | 0 |
| MKHIWA CLINIC | Manzini | 0 | 1 | 1 | 1 | 0 | 1 |
| MKHULAMINI CLINIC | Manzini | 3 | 3 | 6 | 2 | 0 | 2 |
| MLIBA NAZARENE CLINIC | Manzini | 2 | 11 | 13 | 3 | 11 | 14 |
| MOTI CLINIC | Shiselweni | 2 | 14 | 16 | 3 | 6 | 9 |
| MPOLONJENI CLINIC | Lubombo | 0 | 0 | 0 | 0 | 3 | 3 |
| MPULUZI CLINIC | Manzini | 2 | 0 | 2 | 0 | 0 | 0 |
| MSHINGISHINGINI NAZARENE CLINIC | Hhohho | 0 | 0 | 0 | 0 | 1 | 1 |
| MUSI CLINIC | Manzini | 2 | 2 | 4 | 3 | 1 | 4 |
| NATICC CLINIC | Shiselweni | 0 | 0 | 0 | 0 | 0 | 0 |
| NATIONAL PSYCHIATRIC HOSPITAL | Manzini | 1 | 2 | 3 | 2 | 4 | 6 |
| NATIONAL TB HOSPITAL | Manzini | 0 | 0 | 0 | 2 | 1 | 3 |
| NCABANENI CLINIC | Manzini | 8 | 5 | 13 | 9 | 4 | 13 |
| NDZINGENI NAZARENE CLINIC | Hhohho | 0 | 0 | 0 | 1 | 0 | 1 |
| NEW HAVEN CLINIC | Shiselweni | 0 | 0 | 0 | 0 | 1 | 1 |
| NEW THULWANE CLINIC | Lubombo | 1 | 0 | 1 | 1 | 0 | 1 |
| NEW VILLAGE NAZARENE CLINIC | Manzini | 7 | 11 | 18 | 9 | 7 | 16 |
| NEWSTART CLINIC | Manzini | 195 | 173 | 368 | 222 | 103 | 325 |
| NGCULWINI NAZARENE CLINIC | Manzini | 0 | 1 | 1 | 1 | 1 | 2 |
| NGONINI OSSU CLINIC | Manzini | 1 | 0 | 1 | 1 | 0 | 1 |
| NHLAMBENI CLINIC | Manzini | 1 | 1 | 2 | 0 | 2 | 2 |
| NHLANGANO HEALTH CENTRE | Shiselweni | 0 | 0 | 0 | 0 | 3 | 3 |
| NHLETJENI CLINIC | Shiselweni | 0 | 0 | 0 | 1 | 0 | 1 |
| NKHABA CLINIC | Hhohho | 0 | 0 | 0 | 0 | 0 | 0 |
| NKONJWA CLINIC | Lubombo | 0 | 0 | 0 | 0 | 1 | 1 |
| NKWENE CLINIC | Shiselweni | 0 | 0 | 0 | 1 | 2 | 3 |
| NSINGWENI COMMUNITY CLINIC | Hhohho | 0 | 0 | 0 | 0 | 0 | 0 |
| NTFONJENI CLINIC | Hhohho | 0 | 0 | 0 | 0 | 1 | 1 |
| NTSHANINI CLINIC | Shiselweni | 0 | 0 | 0 | 0 | 1 | 1 |
| PHOCWENI CLINIC | Manzini | 2 | 21 | 23 | 2 | 10 | 12 |
| PIGGS PEAK GOVERNMENT HOSPITAL | Hhohho | 0 | 0 | 0 | 1 | 0 | 1 |
| POLICE COLLEGE CLINIC | Manzini | 0 | 0 | 0 | 3 | 0 | 3 |
| PROJECT CANAAN SIDVOKODVO | Manzini | 0 | 0 | 0 | 0 | 0 | 0 |
| RALEIGH FITKIN MEMORIAL HOSPITAL | Manzini | 24 | 68 | 92 | 17 | 28 | 45 |
| SALVATION ARMY CLINIC | Hhohho | 0 | 0 | 0 | 0 | 1 | 1 |
| SAPPI HEALTH CENTRE | Manzini | 1 | 10 | 11 | 3 | 8 | 11 |
| SCUTT JUBILEE (BETHANY) CLINIC | Manzini | 13 | 2 | 15 | 17 | 4 | 21 |
| SHEWULA NAZARENE CLINIC | Lubombo | 0 | 0 | 0 | 2 | 0 | 2 |
| SIGANGENI CLINIC | Hhohho | 0 | 0 | 0 | 1 | 0 | 1 |
| SIGCAWENI NAZARENE CLINIC | Lubombo | 0 | 0 | 0 | 1 | 0 | 1 |
| SIGCINENI CLINIC | Manzini | 0 | 3 | 3 | 0 | 0 | 0 |
| SIGOMBENI RED CROSS CLINIC | Manzini | 3 | 3 | 6 | 1 | 2 | 3 |
| SIMUNYE CLINIC | Lubombo | 0 | 0 | 0 | 0 | 2 | 2 |
| SIPHOCOSINI CLINIC | Hhohho | 0 | 0 | 0 | 0 | 0 | 0 |
| SIPHOFANENI CLINIC | Lubombo | 0 | 0 | 0 | 1 | 4 | 5 |
| SITEKI NAZARENE CLINIC | Lubombo | 0 | 0 | 0 | 1 | 0 | 1 |
| SITEKI PUBLIC HEALTH UNIT | Lubombo | 0 | 0 | 0 | 0 | 1 | 1 |
| SITHOBELA HEALTH CENTRE | Lubombo | 0 | 0 | 0 | 1 | 1 | 2 |
| SOS CLINIC (NHLANGANO) | Shiselweni | 0 | 0 | 0 | 0 | 0 | 0 |
| ST MARYS CLINIC | Hhohho | 0 | 0 | 0 | 1 | 0 | 1 |
| ST THERESA'S CLINIC | Manzini | 0 | 0 | 0 | 1 | 0 | 1 |
| TB CENTRE | Manzini | 0 | 0 | 0 | 0 | 0 | 0 |
| TFOKOTANI CLINIC | Shiselweni | 0 | 0 | 0 | 0 | 1 | 1 |
| THE AIDS INFORMATION & TRAINING CENTRE | Manzini | 19 | 6 | 25 | 19 | 2 | 21 |
| THE LUKE COMMISSION CLINIC | Manzini | 3 | 0 | 3 | 7 | 3 | 10 |
| VUVULANE CLINIC | Lubombo | 0 | 0 | 0 | 0 | 1 | 1 |
| WELLNESS CENTRE CLINIC | Manzini | 0 | 1 | 1 | 1 | 1 | 2 |

^a^Recorded on HIV-test records selected for the study

^b^After HIV diagnosis in community settings in Manzini region, Eswatini
